# Jakartans' Perceptions of Health Care Services

**DOI:** 10.3389/fpubh.2019.00277

**Published:** 2019-09-26

**Authors:** Yasinta Astin Sokang, Alvin Henry Westmaas, Gerjo Kok

**Affiliations:** ^1^Department of Work and Social Psychology, Maastricht University, Maastricht, Netherlands; ^2^Faculty of Psychology, Krida Wacana Christian University, Jakarta, Indonesia; ^3^Faculty of Health Sciences, University of Applied Sciences, Leiden, Netherlands

**Keywords:** health care services, Jakarta, perception, hospitality, Community Health Center, *Puskesmas*

## Abstract

Health Care Services (HCSs) should implement ongoing innovation and continuously improve their quality. However, in evaluating the quality of HCSs, too little attention has been given to the experience of the users concerning the acquired services. This study focused on how the community values the current services in order to improve HCSs in Indonesia, especially in Jakarta. Four focus group discussions were conducted among 45 community members in the Grogol Petamburan sub-district, in Jakarta. Participants were recruited using a convenience sampling and the data were analyzed using a combination of human coding and NVivo-12. Overall, we found that participants had a negative view of the government-mandated Community Health Centers (CHCs) and they preferred to visit private clinics and hospitals over the CHCs. Participants associated CHCs with unfriendly staffs, longer waiting times, shorter opening hours, and crowded visitors. At the same time, participants had a positive view on the affordability of the CHCs. Additionally, we found the reasoning of Jakartans' (i.e., the citizens of Jakarta city) on using self- and traditional treatments before visiting HCSs and they also expressed the need for psychological services at CHCs. The discussion focuses on the results as feedback on how the government and health care providers may facilitate the community's needs in providing HCSs in Indonesia, especially Jakarta. In brief, we recommend the policy-makers to improve the hospitality of the staff members and the quality of the medical equipment; also, to provide psychological services at CHCs. These efforts need to be done while paying more attention to the cultural aspects of medicinal uses.

## Introduction

Improving health through improving health services and health promotion activities is currently one of the six priority programs of the Indonesian government (1, 2). However, the government has encountered many obstacles in running their health-improvement programs, especially in Jakarta. Specifically, they have had problems with limited human resources, inadequate health facilities, unhealthy behavior of the citizens, the population density, and poverty (1, 3). Together with the heterogeneous nature of the citizens of Jakarta, this situation creates a complex demand for health care providers (1). To overcome these obstacles, the Health Ministry periodically evaluates the quality of Health Care Services (HCSs) by conducting an accreditation (4). This accreditation emphasizes the development of the quality and performance of HCSs through continuous improvements of its management system; its service and program delivery system; and its implementation of risk management.

The health care system in Indonesia is a combination of public and private health providers. The public health provider is the Indonesian government; in this case, the Ministry of Health at the national level, provincial governments at the provincial level, and the district/municipality governments for district level. The private health providers manage by not-for-profit or for-profit providers, and individual doctors and midwives who have a private clinic. The financial support of health centers is the responsibility of the provider. The health care user can pay for health services using personal funds, or by using insurance. Currently the Indonesian government promotes the use of a universal health insurance. With the insurance, the user could get a cheaper or even free service by visiting Community Health Center [CHC; Indonesian: *Pusat Kesehatan Masyarakat (Puskesmas)*] (5).

To support the health improvement program, one needs to conduct a thorough evaluation of the current state of the available HCSs. A more inclusive approach to said HCSs evaluation would include the perspectives of its users (6); this inclusion would help stakeholders to understand the health care needs, demands, and interest of the communities better (7, 8). Here, the users are considered as experts (9).

As a first step to evaluate the perspectives of health care users as experts, we conducted a study to understand Jakartans' (i.e., the citizens of Jakarta city) perceptions regarding health and sickness (10). We found that the Jakartans not only see health as a physical state, but also as psychological, spiritual, and the ability to perform daily activities productively states. We also found the importance of investigating the perspectives of communities to fully understand their beliefs, values, and needs, in order to provide health care services that fit the demands of the communities. Our study suggests that incorporating the perspectives of users is an important key to improve the quality of HCSs.

In spite of the importance of understanding the perspectives of health care users, only little research has been done on the perceptions and expectations of health care service users in Indonesia, especially in Jakarta. In the absence of local empirical findings, the Jakarta government and health care providers are more likely to incorporate the typical Western approaches on health programs, which emphasize the biomedical perspective, to improve the quality of HCSs in Jakarta. Furthermore, the aforementioned periodical accreditation does not take into account the users' experiences in evaluating the quality of HCSs. The lack of information on the experiences of the community of existing HCSs users makes it difficult, if not impossible for policy-makers to identify and satisfy the needs of the community.

Therefore, the main goal of the current study was to evaluate how the health care users in Jakarta perceive the quality of its HCSs. The perspectives of users would provide critical information which can be used to evaluate to what extent the available HCSs meet the expectations of its users. The resulting data could provide a recommendation for the government, health care providers, and health promoters in Jakarta to formulate health programs and policies which fits the needs of the local communities.

## Methods

As a framework, this study used the Intervention Mapping (IM) approach which consists of six steps. The approach focuses on using theory and evidence as a solid basis for building a health program planning (11); thus provides clear guideline descriptions of the planning and designing, developing, implementing, and evaluating the program objectives (12, 13). In this current study, we applied the first step of IM which emphasizes the importance of needs assessment to understand the target community, in terms of behavior and environmental determinant, including the characteristics of the community. Using this approach, we aimed to understand the health care users' perception on the quality of HCSs, while paying attention to culture and social aspects of the issue, including the needs of the community.

### Participants

Data was collected in four focus group discussions (FGDs) led by the first author in October 2017. In total, 45 Jakartans from various ages (20–66 y/o), from low and middle economic status (±35–320 USD/month), and from various educational levels (elementary school–bachelor degree) participated (18 males; 27 females). They were recruited using a convenience sampling and have given written informed consent to participate in the study. The participants were the member of the Grogol Petamburan sub-district, in West Jakarta Municipality, Special Capital Region of Jakarta. The Special Capital Region of Jakarta consists of five municipalities with the similar characteristics of the community member; as they come from various backgrounds. Of the participants, 30 were native Jakartans or had identified themselves as Jakartans, and 15 were from other areas in Indonesia. The participants have equal access to CHC (private), clinic, and hospital; although not all of them applied for national insurance. Despite the various demographic backgrounds, there were no systematic differences regarding our respondent's opinions on the quality of HCSs in Jakarta. Therefore, the results and discussion will focus on the participants' perspectives regardless of their demographics.

### Materials and Procedures

Data were collected in FGDs to acknowledge people's experience, and to capture and explore people's ideas and perceptions on the topic at hand (14, 15). The FGDs were conducted in the Indonesian language, in locations that were convenient for the respondents, and lasted for 68 min on average (min 53′-max 90′).

Four research assistants (graduate bachelor students in psychology) were trained to answer any questions potential respondents might have during the FGDs, to observe the FGDs and make notes, and to conduct data analysis together with the first author. Prior to data collection, the assistants acquired an approval letter from the local village office to conduct the study. Upon receiving the approval letter, they came to different local communities in the Grogol Petamburan sub-district during the afternoon (when people usually gather in front of their houses), they asked people whether they would like to participate in the study, and they provided the opportunities for people to ask questions regarding the study. If potential participants agreed to participate in the study, the assistants provided several options for the FGD venue. Upon agreement, the assistants sent a formal invitation to the participants to participate in the FGD. In the invitation letter, we again explained the purpose of the FGD, the procedures of the FGD, the outline of the FGD questions, and the place and the duration of FGD. A statement of confidentiality letter was enclosed in the invitation letter. After participants had agreed to participate, they received the informed consent form. With the informed consent, there was information about the study purpose, and agreement of using video and voice recorder during the FGD. This is followed by the information regarding the members of the research team (name, faculty, university, and email address), and a questionnaire for participants to indicate their demographics, such as their sex, age, education level, job, income/allowance per month, and origin.

The FGDs were opened with a welcome, and an introductory round of the team members and the participants. The first author, who acted as a facilitator then described the general purpose of the meeting, the data confidentiality, and ensured that participants filled and signed the written informed consent form. Next, the facilitator explained the FGD procedure and established the ground rules of the FGD. Furthermore, the facilitator introduced the topic of discussion and started the discussion. The questions of FGD were presented as open-endedly as possible. The guideline questions were “If you are sick, where would you go?”; “What are your expectations when you went there?”; “How do they treat you? Does it fit your expectation?”. Unclear ideas or opinions were clarified by open and probing questions, such as “Can you explain that?”; “Why?”; “Can you give an example?”. Before closing the FGD, the facilitator summarized the discussion and distributed a short survey among the participants to ask whether the participants wanted to add something to the summary. Afterwards, the facilitator closed the discussion by thanking the participants for their contributions.

### Analysis

The FGDs were recorded and transcribed verbatim. Due to the lack of information on Jakartans' perceptions of HCSs, a content analysis with an inductive approach was applied to create categories of the responses (16, 17). A combination of human coding and NVivo-12 was used to analyze the data. Human coding was applied to the Indonesian language version of the data. Open coding and categorizations were performed by the research assistants under the supervision of the first author. The texts were read repeatedly by the assistants to identify meaningful words or sentences. Words and sentences with similar meaning were grouped under one category (18). The saturation of the data was achieved when there was no new category and theme emerged from the texts. This approach is also called the inductive thematic saturation (19). Later, the transcriptions were translated into English and analyzed using NVivo-12 by the authors. This process was done to ensure both human coding and NVivo-12 analyses would fit with one another.

## Results

Although, we describe the participants' views toward HCSs on separate themes, it is important to keep in mind that these themes were not entirely independent from one to another. The themes portrayed the participants' experience in using HCSs in Jakarta. Participants claimed to visit Community Health Center [CHC; Indonesian: *Pusat Kesehatan Masyarakat (Puskesmas)*] and (private) clinic more often than hospital, so only a few statements describe their experience in hospitals.

### Hospitality of the Staff Members

The hospitality of the staff members is the most emerging theme. This theme includes the durations of waiting time (before service) and for medical examinations. There were two topics that concern participants the most. Firstly, they perceived CHC staffs as “*less friendly*” (female, 20 y/o), “*cruel*,” “*rude*,” and made them “*feel distinct*” (female, 29 y/o). Secondly, the waiting time in CHCs was long due to a large number of people who typically visit the CHC during its opening hours. One participant stated that whether she “*wanted or not, as long as (the CHC is) open*” (female, 35 y/o), she would wait to get her treatments. However, after the patients got treated, the general practitioners only asked brief questions and they “*immediately gave the solution, gave medications*” (female, 41 y/o). Participants believe that the general practitioner treated them hastily because “*time is expensive*” (female, 41 y/o) for the general practitioner. This experience created a negative perception about the CHC.

Compared to the CHCs, participants preferred to get medical treatments at private clinics when they got ill. They perceived staff members of the private clinics as more friendly and welcoming. Additionally, the queue was shorter; therefore, they can get examined immediately. Likewise, one participant stated that the private clinics and hospitals were the best because the medical information was “*more specific; any kinds of complaints were more heard*,” so she was “*more satisfied*” (female, 37 y/o).

### Quality of the Services

The quality of HCSs' services includes the quality of its facilities and medical devices. With regard to CHCs, participants believed that “*at most the medicines were generic medicines; even patented medicines were not there*” (male, 20 y/o). Generic medicines were perceived to be less effective because the patients think that he/she “*Needed to come back, took longer to recover*” (female, 41 y/o). Participants assumed that CHCs only provide medicines for minor illnesses, such as coughs, colds, and fever. Some participants viewed CHC in a negative way, because “*on average the CHC's equipment, mostly, was old; maybe because it's never replaced*” (male, 20 y/o). On the other hand, the participants realized that it happened because the CHCs “*only relied on subsidies from the government*” (male, 20 y/o).

Meanwhile, participants were more confident with the quality of medicines at private clinics. One participant shared that “*in (the) CHC, (I) had treatment; (after) three days (I was) not getting better, (in spite of) the medicine was given. After I run (went) to the clinic, thank God, (with) this medicine, (I got) healed. So, I choose clinics*” (male, 32 y/o). Participants willingly pay more, because they believe that the clinics put quality first by providing specialists. On the contrary, a lady who has a baby found “*(the vaccination) from the CHC likely to be trustable; it comes directly from the Minister of Health, from the Health Office. The truth is, the clinics are the one that we are really wary of… We do not know where their vaccine from, where it takes from*” (female, 35 y/o). As a side note, this statement might be biased by news about bootleg-vaccines that came out prior to the FGDs.

### Service Fee

Participants had a positive view on CHCs concerning their service fee. The inexpensive cost of medicines and treatment made participants go to the CHC because “*if (I use) national health insurance, (it's) free, even if (I) pay, only three thousand five thousand (IDR;* ±*0.21 USD)*” (female, 41 y/o). To receive a better treatment at larger hospitals, participants would “*go to the CHC to only ask for a referral letter; that's why I always go to the CHC*” (male, 21 y/o).

A more negative view was expressed toward the private clinics and hospitals' service fee. Most participants agreed that “*if (I) go to the clinic, (you) pay a little more expensive*” (male, 46 y/o), because “*it will cost a bit different from the CHC; the medicines' quality is definitely different*” (male, 46 y/o). They went to the hospital only if they got a referral letter from the CHC, so that the treatment will be free or cheaper than the regular treatment without a referral letter. One participant mentioned that her brother is a general practitioner in a hospital “*so, if my family gets a severe illness, directly to his hospital; my brother makes the referral (letter)*” (female, 21 y/o). Nevertheless, a lady who has a baby disclosed that even though the hospital is expensive, she will use the service again if her child is sick.

### Opening Hours and Distance

Participants tend to have a negative perception of CHCs due to the short opening hours. Moreover, the long distances made them less motivated to visit the CHC as they need extra time and funds.

In contrast, the private clinics are often open for 24 h, and they can be relatively close. The availability and distance allowed participants to “*go to the clinic because the clinic is 24 hours. Once, around one o'clock or two a.m. (I was) sick and went straight there (the clinic)*” (female, 21 y/o). They were able to visit the clinic on a walking distance, so they did not need extra money. Private hospitals are also generally open for 24 h. However, “*(it's) tiring to go to the hospital, (because it's) far, (and) moreover (it's) expensive*” (female, 42 y/o).

### Self- and Traditional Treatments

In addition to their views on HCSs, most participants also indicated that they preferred to do self-treatment before going to the HCSs when they were sick. One participant stated “*If sick, (I) usually (buy) medicine (from small) stall first; later, if still sick after a few days, (I) go to the CHC*” (male, 36 y/o). Participants indicated that they would take a rest at home and ignore their pain while they observed how their sickness develops. If their condition improves, they would not seek additional treatments. They will visit a general practitioner if their illness got worse.

Some participants stated that they prefer the traditional medicine, such as herbal drinks (Indonesian: *jamu*) and they applied the local practice of coining (Indonesian: *kerokan*). Participants choose to do self-treatments in cases in which their sickness is a minor one, for example, when they got a cold (Indonesian: *masuk angin*). In this case, there was no need for a special treatment, such as going to the general practitioner. One participant said “*if I get sick… mostly buy medicine or look for traditional medicine. Because traditional medicine, it is herbal and it is passed down, given to us. From our grandfather's era the medicine is the same. Indeed, we already know it's more powerful than the medicines we purchase in pharmacies with whatever substances in it. I prefer traditional medicine, which (is) definitely herbal; it's an inheritance from long time ago*” (male, 22 y/o).

### The Need for Psychological Services

Lastly, participants expressed their need for psychological services at HCSs. They mentioned the need for counseling for their daily life problems, for child development issues, and to get additional information about parenting skills, such as on guiding their children to learn more efficiently and to deal with teenagers.

In one of the communities, there were residents with severe mental disorders. The participants indicated that they had proposed to bring the patient to a mental institution, but the family of the patient declined the idea. The participants chose to be quiet in this case, because they have limited knowledge about mental health and were worried that they would offend the family. One participant stated “*the psychologists (are) special(ist), (they know) what to ask the patient. So, (they) already know, oh, I want to ask this and this, can (do/say) this. For us, sometimes (when we) ask the person get angry, we will stop*” (female, 57 y/o).

## Discussion

The aim of the present study was to understand the perceptions of Jakartans in regard to the quality of available HCSs. A better understanding of Jakartans' perceptions of HCSs would contribute to the evaluation of to what extent the available HCSs meet the expectations of the users. This information can help the government, health care providers, and health promoters to formulate health policies and programs for Jakartans while taking the community's point of view into account. Overall, our findings indicate that the HCSs, currently available in Jakarta, do not provide services that meet the values that are considered important by the communities. This leads to the tendency of the participants to do self-treatment before going to the health care centers.

As stated before, we discussed the themes separately, however, the readers may notice that they were not entirely independent from one to another. The hospitality of the staff members is the most expressed theme by our participants in relation to the perception of HCSs in Jakarta. This theme includes the length of waiting time and for medical examination. At the same time, the waiting time and the duration of medical examination links with opening hours and distance, and with the quality of the facilities and medical devices.

Previous studies have found that the hospitality of health care services staff members is important to shape a positive image for the users (20). Users, in this case the patient and her/his family, prefer friendly staff because they are approachable and easy to make contact with. Users perceived these characteristics as sympathetic and caring; so that, they trust the staff members to take care of them or their family member (21). Caring and friendly services have also been found to be associated with the increase of user satisfaction (8, 22). As a result, the intention to visit the same HCSs and to use the services of HCSs will increase (23). In line with these findings, our participants prioritized hospitality over service fee; they are willing to pay more as long as they get friendly services from the staffs. In our case, the participants chose private clinics over CHCs because they experienced unfriendly services at CHC. As a note; it is more expensive to go to the clinics. The staffs probably get paid better than the CHC's staff and are therefore friendlier.

Previous researchers have found that waiting time affects people's perceptions about the given services and in turn, patients would choose to get a treatment from another health care provider the next time (24, 25). However, the HCS in Jakarta, especially CHCs, faces challenges to implement shorter waiting times. In Jakarta, there were too many CHC patients while there were too few general practitioners (3). The use of the national health insurance has resulted in each general practitioner to treat about 100 patients each day; consequently, each patient only had about 4 min for the overall treatment (26). In addition, the duration of medical examination is limited due to the shorter service hours (i.e., 07.30 a.m.−4.00 p.m.; break 12.00–1.00 p.m.) (27). The outcomes to the limited opening hour at CHCs with an overload of patients are long waiting times and short examination times, as experienced by our participants. Consequently, our participants expressed a more negative view toward the CHCs' services and they chose private clinics and hospitals over CHCs. This preference might have also been influenced by the fact that most private clinics and hospitals provide 24-h services.

The use of modern and appropriate technology is important for the users of HCS (18, 21). Our participants admitted that they did not go to the CHC to get a medical treatment because they perceived the medicines to be of low quality, and the equipment to be old and inadequate. For them, it is useless to go to the CHC because for in-depth medical examinations, they would be referred to a hospital or a laboratory upon their CHC visit anyway. This view was in contrary to the statement of one lady who has a baby, she was confident with the quality of the vaccination provided by the CHC, and doubted it when it came from private clinics. This belief might be related to the news about bootleg vaccines that came into view prior to the FGD. Even though the Indonesian Health Ministry published the list of convicted hospitals and clinics (28), our participant might have held her doubt.

A low cost is one of the reasons why people use health facilities (21). In Indonesia, the government promotes the use of the national health insurance since 2011, which makes medical treatment and medicines cheaper, or even free of charge (4). However, to get the benefit of the national insurance, one must visit the CHC. If the CHC's general practitioner refers the patient to another HCS, the treatment and medicines will be covered fully or partially (29). This benefit leads to a positive view of the CHC by our participants; they prefer to go to the CHC rather than to private clinics or hospitals to get the benefit of lower cost. There were cases in which the participants went only to CHC to get the referral letter, to get a better treatment at a bigger hospital. Some patients even force the general practitioner to give them the referral letter because the CHCs' service is considered to be insufficient (30). In contrast, some participants directly go to the private clinics or hospitals even though it is expensive. They did not mind paying more because friendly and comprehensive services are the most important aspects for the participants.

In this study, the different HCSs mentioned by the participants had their advantages and weaknesses. In some cases, the participants did self-treatment because buying medicine in stalls is cheaper than going to a nearby clinic. If they did not get better, then they will visit either the CHC or the clinic. Self- and traditional treatments are used because it is a hereditary tradition, and people believe in its effectiveness (31–33). Most of our participants thought that in cases in which their sickness was a minor illness, they did not require a medical treatment from a general practitioner or a medicine subscription. On the other hand, self-treatments might cause the illness to get worse and the CHC's medicines might no longer have an adequate efficacy to treat this developed illness (34, 35). Traditional medicine is not prohibited by the Indonesian government as stated in the Act of the Republic of Indonesia number 36 of 2009 about Health (36). It emphasized on Regulation of Ministry of Health Republic of Indonesia number HK.01.07/MENKES/187/2017 (37) which underlined that citizens can use traditional medicine as long as they consult with a health care worker, use it rationally by following the instructions without neglecting the general practitioner's treatments or prescriptions. However, this information is unknown to most people, which might be due to the different understandings about self- and traditional treatments between patients and health care providers (34, 38). The mismatches in understanding might result in discouraging patients to discuss the use of self- and traditional treatments with their general practitioners (34, 39).

The needs of psychological services appeared as an additional information. However, it confirms the findings from our previous study (10) which showed that a psychological need is of significant value for the community to be healthy. Psychological health is important, both in relation to physical aspects as well as in social aspects, and one's ability to carry out daily activities. In contrary, the presence of psychologists at CHCs in Jakarta is limited. Psychological services are only available in private practices, hospitals and clinics (40). Turning to Jogjakarta, one of the provinces of Indonesia, psychological practices have been provided in CHCs since 2010. CHC's psychologists detect early mental disorders in the community and they conduct prevention programs for mental health (41). Our previous study showed that participants needed psychological services, but the service was not available at the CHC. The participants expect the CHC to provide psychological services in order to help them deal with daily problems and severe mental disorders.

The findings of this study provide insights on issues that are considered important by the community, such as staff's hospitality in HCS. The willingness of the participants to pay more in order to get friendly services shows the value of friendly services to the community. Friendly services make people comfortable to visit HCS. Beside the service improvements, the medical equipment quality used at HCS also one of the community's concerns. The use of modern and good-quality equipment would increase the community's trust in HCS. In addition to medical equipment, the quality of the medicines also needs to be improved so that people can get a more efficient medical treatment at the CHCs.

The government should pay more attention to the cultural aspects of health, such as taken into account the use of traditional medicines. Valid information with regard to how traditional medicines are regulated needs to be provided, both to health care providers and the public. Dissemination of the government regulations, such as regulation number HK.01.07/MENKES/187/2017 about the ingredients of traditional medicines in Indonesia is necessary so that people become aware of the proper instructions to take traditional medicine. By addressing the issue with traditional medicine, future patients might be more willing to openly discuss the use of traditional medicines with formal health workers and in turn, this interaction would help to increase the overall level of health in the community. The medical team should be aware that their negative reactions toward traditional treatments could influence how their patients share their alternative medicine consumptions and seek medical treatments in the HCSs. Another effort to consider is to involve traditional medicine practitioners in HCSs. This approach has been implemented in other countries with positive results (42–44).

There are strengths and limitations of our study that are worth mentioning. Firstly, to the best of our knowledge, the data on how the Jakartans value the current HCSs are rare, particularly the data taken with a qualitative approach. Secondly, the generalizability of the present results may be limited due to the small number of participants. At the same time, our data includes responses from participants from different ages and educational levels. On the other hand, this study may be subject to a potential bias due to the sampling technique used. Using this research as an initial step, we suggest future studies to conduct multi-site studies with quantitative or mixed methods involving larger samples from other areas of Jakarta, with more diverse demographic backgrounds. The study could explore the other themes or issues that might emerge from a broader sample, or focus on the perception or experience of specific age- or sex-group. Another study might explore the overall satisfaction of the community, as well as evaluating the treatment effectiveness.

## Conclusions

This study explored the Jakartans' perceptions on HCSs, focusing on the hospitality of the staff members and the services of staff members and general practitioners. Overall, our participants expressed their preference to private clinics and hospitals over the government-mandated CHCs. The CHCs had long waiting time and short opening hours, while private clinics had friendlier services, shorter waiting time, and longer opening hours, even though they had to pay more for the services. At the same time, the participants expressed a more positive view on the CHCs with regard to their service fees, which were affordable or even free under the national health insurance coverage. In summary, these results show that the current services offered by HCSs in Jakarta do not meet the needs of their communities. To alleviate this problem, we suggest the CHCs to take into account the needs and values of the community, including, but not limited to, incorporating a more open view on traditional medicine and providing psychological services. We hope that these recommendations would be taken into account in the future health-related policies and programs in Jakarta.

## Data Availability Statement

The raw data supporting the conclusions of this manuscript will be made available by the authors, without undue reservation, to any qualified researcher.

## Ethics Statement

The studies involving human participants were reviewed and approved by The Ethics Review Committee of the School of Psychology and Neuroscience, Maastricht University (Reference number: ERCPN 04-09-2012_S21), and the Grogol Petamburan sub-district official on the letter number 424/UKKW/FPsi/Prodi/X/2017. The participants provided their written informed consent to participate in this study.

## Author Contributions

YS performed the study design, data collections, and data analysis, including the coding, categorizing and drafting the original manuscript. AW performed the data analysis and reviewed the original manuscript. GK provided feedbacks for the analysis and reviewed the original manuscript. All authors read and confirmed the final version of the manuscript.

### Conflict of Interest

The authors declare that the research was conducted in the absence of any commercial or financial relationships that could be construed as a potential conflict of interest.
